# Serum Level of Vitamin D in Patients with Salivary Gland Tumors 

**DOI:** 10.22038/IJORL.2023.69088.3346

**Published:** 2023-09

**Authors:** Sedigheh Moayedi, Bijan Khademi, Mahyar Malekzadeh, Golnoush Farzinnia, Zohreh Jaafari-Ashkavandi

**Affiliations:** 1 *Department of Orthodontics, School of Dentistry, Shiraz University of Medical Sciences, Shiraz, Iran.*; 2 *Otolaryngology Research Center, School of Medicine, Shiraz University of Medical Sciences, Shiraz, Iran.*; 3 *Institute for Cancer Research, School of Medicine, Shiraz University of Medical Sciences, Shiraz, Iran. *; 4 *Oral and Dental Disease Research Center, School of Dentistry, Shiraz University of Medical Sciences, Shiraz, Iran.*; 5 *Department of Oral and Maxillofacial Pathology, School of Dentistry, Shiraz University of Medical Sciences, Shiraz, Iran.*

**Keywords:** Cancer, Mucoepidermoid carcinoma, Pleomorphic adenoma, Salivary gland, Vitamin D

## Abstract

**Introduction:**

The active vitamin D metabolites have anticancer effects on many human neoplasms. The vitamin D receptors have been detected in salivary glands tissue. This study aimed to evaluate the serum level of vitamin D in patients with malignant and benign salivary gland tumors.

**Materials and Methods:**

In this retrospective and cross-sectional study, 151 participants, including 42 patients with benign, 42 malignant salivary gland tumors, and 67 healthy subjects, participated. The serum level of vitamin D was measured using an enzyme-linked immunosorbent assay (ELISA).

**Results:**

The mean serum level of vitamin D was 42.7 ng/mL in patients with benign tumors, 40 ng/mL in malignant tumors, and 36.7 ng/mL in the control group. There was no significant difference between the mean vitamin D level and vitamin D status in patients with salivary gland tumors and normal controls (P=0.2). There was a significant positive correlation between vitamin D level and age in the control group (P=0.04).

**Conclusions:**

The results showed a high prevalence rate of vitamin D deficiency/insufficiency in salivary gland tumors and normal subjects, with no significant difference. Therefore, the serum level of vitamin D might not play a significant role in the pathogenesis of these tumors, similar to many human cancers. However, further prospective studies are recommended focusing on specific tumors and considering other interventional factors.

## Introduction

Salivary gland tumors (SGT) account for approximately 3-5% of head and neck tumors. They usually arise from the parotid gland, and a high percentage of them are benign. Pleomorphic adenoma (PA) is the most common benign SGT, and mucoepidermoid carcinoma (MEC) and adenoid cystic carcinoma (ADCC) are the most common malignancies in this area (1). Despite the many head and neck tumors, the exact reason for SGT is unknown. Tumors have multifactorial etiology; some have environmental factors contributing to the formation of these epithelial tumors. The role of alcohol and tobacco consumption is controversial (2). It is also suggested that long-term use of cell phones and some viral infections increase the risk of SGTs (3,4). The other possible factors triggering these tumors are diagnostic and therapeutic radiotherapy and dietary factors (5). Moreover, specific gene mutations are associated with some SGTs (6). 

Preclinical studies suggested that the active metabolite of 1,25-dihydroxyvitamin D3 (1,25 (OH)2D3), known as calcitriol, or vitamin D analogs, has anticancer properties due to their anti-proliferative, anti-angiogenic, and apoptotic activities (7). The active form regulates gene transcription by attaching to nuclear vitamin D receptors (VDRs). Many studies have revealed a connection between VDR genotype and the risk of cancer development (8).

Vitamin D3 increases the expression of the Transforming Growth Factor-β1 and β2 (TGF- β1 and β2) genes and downregulates the expression of the prostaglandin E2 (PGE2) gene, leading to a decrease in the risk of breast cancer incidence (9). 

A study showed that the higher vitamin D serum levels were associated with enhanced the incidence of prostate cancer (10). Epidemiologic studies have shown that serum level of vitamin D is an important factor in developing different cancers, such as colorectal (11), larynx and oropharyngeal (12), breast (9) cancers, and reproductive tumors (13). 

Although different studies have investigated this issue, there is little information available about the serum level of vitamin D in patients with SGTs. Previous studies detected VDR in salivary glands (14). Moreover, the reduced tumor extension was shown in the ADCC cell line of salivary gland tumors and a tumor model after 1, 25 D3 treatments (15). In the present study, the serum level of vitamin D has been evaluated in patients with benign and malignant SGTs compared to normal subjects.

## Materials and Methods

This research was approved by the Medical Ethics Committee of Shiraz University of Medical Sciences (IR.SUMS.REC.1397.249). In addition, written informed consent forms were obtained from all the participants. In this cross-sectional study, 84 patients with benign and malignant SGTs referred to Khalili hospital affiliated to Shiraz University of Medical Sciences, and 67 healthy volunteers participated, from November 2018 to November 2019. 

SGTs included 38 subjects with PA and 4 other types of benign tumors, 42 with malignant tumors (16 MEC, 11 ADCC, 7 squamous cell carcinoma (SCC), and 8 other types of carcinomas).

Patients with a history of previous anticancer treatments, disease recurrence, or those who had recently (the last two months) taken complementary and vitamin D medication, as well as the presence of other inflammatory or systemic diseases and also diseases those alter vitamin D serum levels such as thyroid or parathyroid diseases and hyperparathyroidism, were excluded from the study. The control group was age and gender-matched healthy subjects with cancer patients.

From each patient and the normal control group, a 2 cc serum sample was prepared and stored at -70°C until further analysis. Vitamin D concentration was measured using a sandwich ELISA test with a Monobind ELISA kit. The serum levels <20, 20-29, 30-100, and > 100 ng/mL were considered as vitamin D deficiency, insufficiency, normal, and toxic, respectively (16).

*Statistical Analysis:*

Data were analyzed using SPSS software. The Kruskal-Wallis, Mann-Whitney, ANOVA, Chi-square, and Spearman’s rank correlation tests were used to compare the mean level of vitamin D and vitamin D status between groups. P <0.05 values were considered to be statistically significant.

## Results

Baseline data regarding patients’ age, gender, and tumor stage are shown in Table1. According to one-way ANOVA and Chi-square tests, the participants were homogeneous regarding age and gender (P> 0.05). 

**Table 1:** Baseline data of all study groups

**Groups (N)**	**Age (Mean±SD)** **Min-Max**	**Gender** **F/M**	**Patients ‘Clinical Stage (N)** **I,II,III,IV**
Benign tumors(42)	45±11.8 26-70	27/15	-
Malignant tumors(42)	53±19.2 12-83	19/23	8,12,10,12
Control(67)	50±13 22-86	30/37	-
Total(151)	49.5±14.9 12-86	76/75	8,12,10,12
			

The mean of vitamin D levels was 36.7 ng/mL in the control group, 42.7 ng/mL in patients with benign, and 40 ng/mL in patients with malignant tumors. The Kruskal–Wallis test showed no significant difference in the mean serum level of vitamin D among the groups (P=0.2). Tables 2 and 3 show the mean, maximum, minimum concentrations of vitamin D, and vitamin D status in all groups. Vitamin D deficiency/insufficiency was detected in 49.2% of the subjects in the control group, 30.9 % of subjects with benign, and 42.8% of subjects with malignant tumors. The Chi-square test showed no significant differences in vitamin D levels amongst groups (P=0.1) (Table 3).

**Table 2:** Mean of vitamin D among patients and control groups

**Groups:(n)**	**Sub Groups**	**Vitamin D (ng/mL)** **Mean±SD**	**Vitamin D** **(Min-Max)**	P- value*
Benign tumors:	PA(38) Other(4)	42.7±28.1 43±29.3 40.5±20.3	8.6-101.8	0.2
Malignant tumors:	ADCC (11) MEC (16) SCC (7) Other (8)	40±28.9 40.2±24.6 43.2±31.8 37.3±34.8 35.9±22.7	4.9-102.7 18.6±78.2 9.4±102.7 4.9±99.4 13.7±84.3	
Control	_	36.7±23.4	3.8-107.3	
Total	_	39±23.8	3.8-102.7	

*According to Kruskal–Wallis test

**Table 3:** Vitamin D status in patients and control groups

**Groups**	**Deficiency**	**Insufficiency**	**Normal**	P-value*
Benign tumors	5(11.9%)	8(19%)	29(69%)	0.1
Malignant tumors	10 (23.8%)	8(19%)	24(57.2%)	
Control	24(35.82%)	9(13.43%)	34(50.7%)	
Total	39(25.82%)	25(16.55%)	87(57.61%)	

*According to Chi-square test

**Fig 1:**
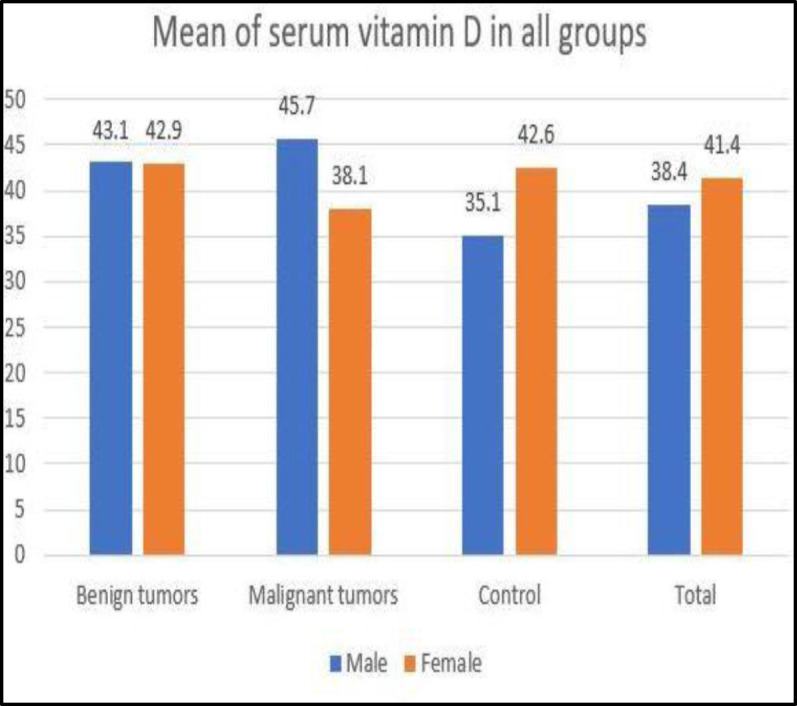
The mean of serum vitamin D in all groups based on gender

The correlation between patients’ age with vitamin D levels was evaluated using Spearman’s rank correlation test. It showed that the vitamin D level was not affected by the patients’ age with benign tumors (P=0.1, rho=0.22) and malignant tumors (P=0.4, rho=0.25). However, there was a significant direct correlation between vitamin D level and age in the control group (P=0.04, rho=0.25). A higher level of vitamin D was observed in older individuals. Mann-Whitney test did not show any significant difference between vitamin D levels in males and females. (P=0.43). Details are shown in figure 1. 

## Discussion

  Present study results showed that vitamin D deficiency/insufficiency was frequently found in the patients with benign or malignant SGTs and the age and gender-matched healthy subjects, with no significant difference.

Several studies investigated the correlation between vitamin D concentration and the risk of malignancies, obtaining different and even controversial results. It seems that the normal or abnormal level of vitamin D in patients is more important than the mean concentration of vitamin in the sera. In this study, 31% of patients with benign tumors, 43% with malignant tumors, and 49% of the control subjects showed vitamin D deficiency/insufficiency, with no statistically significant difference. Similarly, in another study, 55% of non-Hodgkin’s lymphoma patients had a normal vitamin D status, and 44% of them were detected to have vitamin D deficiency (17). 

However, in one study, 65% of patients with head and neck cancer were revealed to have vitamin D deficiency and insufficiency (18). In a study by Calmarza et al. on patients with urologic, colorectal, and head and neck cancer, 80% of them had vitamin D deficiency/insufficiency, which was more significant in patients with head and neck cancers (19). This deficiency has been observed in patients with recently diagnosed advanced pancreatic and colon cancer, especially in people with dark complexion and women (20,21). 

Our result also showed a high prevalence rate of vitamin D deficiency in the control group. Vitamin D insufficiency is quite common amongst people due to increased use of sunblock, increased indoor activities, and skin covered by clothing (22). Tabrizi et al. evaluated 48 systematic reviews and 18531 subjects, stating that the prevalence of vitamin D deficiency in Iranians was significant and varied in different geographical regions, concluding that appropriate programs must be implemented. In the aforementioned study, the prevalence of vitamin D deficiency in men, women, and pregnant women was 45%, 62%, and 60 %, respectively (22).

It seems that cultural factors, type of clothing, and skin full coverage outside, especially in women, and also nutritional factors, such as low consumption of seafood in the Iranians, play a role in reducing the vitamin D level. This matter should be considered in general health programs, duo to the significant roles of vitamin D in life. 

This article was a primary study to evaluate the serum levels of vitamin D in patients with SGTs. The current study did not reveal a significant difference between tumor patients and normal subjects. These findings are in line with a study by Hannah Arem et al. who did not observe any relationship between the serum 25(OH)D concentration and the risk of larynx and oropharynx cancers. In the aforementioned study, the mean vitamin D concentration, as well as the normal or abnormal level of vitamin D in patients and control group, were the same, and the findings did not support the hypothesis that the higher serum level of vitamin D reduces the risk of head and neck SCC (12). McCullough et al. also did not find any significant relationship between the serum level of 25(OH)D and the risk of breast cancer after menopause (23).

Based on the results, it seems that vitamin D might not have a significant role in SGT pathogenesis. Huang et al. showed that vitamin D could reduce proliferation rate and cisplatin resistance in the ADCC cell line. 

Active metabolites of vitamin inhibited the tumor invasion and migration in vivo. They found that NF-κB and glutathione peroxidase 1 (GPX1) were reduced after cell treatment with 1,25 (OH) 2D3 (15). Different results provided in the present study might be due to the fact that we examined different types of malignant tumors. Each malignant tumor might be different from others in terms of vitamin D serum level, such as MEC that was considerably different from other cancers. The vitamin D values might change due to some conditions, such as smoking, sampling season, body mass index (BMI), high sun exposures, exercise, and skin complexion (23). All of these factors may have influenced our findings. Since the results of studies are controversial regarding factors, such as smoking and sampling season; hence, it is better to examine the level of vitamin D in different sessions for more accurate results. However, due to the rarity of the tumors, especially the malignant ones, this examination was impossible in this study. 

If patients take vitamin D supplements in two months, it can interfere with the results. Moreover, patients might change their diet or lifestyle after realizing the disease symptoms. Some researchers have stated that a lower post- diagnostic vitamin D levels might be the consequence of the disease rather than the reason for cancer, which might be due to some changes in lifestyle, such as less physical activity, sun exposure, food intake, or systemic effects of cancer itself (8).

The result of a cohort study on the patients with pancreatic cancer did not support the hypothesis that 25(OH)2D) concentration plays a protective role against pancreatic cancer (24). Xue et al. also confirmed this finding and stated that the higher concentration of vitamin D increased the risk of prostate cancer (10). Moreover, park et al. showed that oral vitamin D intake had a direct positive relationship with the risk of basal cell carcinoma. Their study did not support the beneficial role of oral vitamin D intake in preventing skin cancer (25).

Many in vitro and in vivo studies have shown that vitamin D has anti-proliferative, pro-apoptotic, and anti-angiogenic properties. Calcitriol treatments and many cytotoxic medications have synergic or at least coordinating effects (26). 

Vitamin D3 upregulates the expression of TGF-β1 and TGF-β2 genes and reduces the PGE2 expression and, eventually, the risk of breast cancer development (21). Also, in one study, serum 25(OH)D was showed to have a protective effect on colon cancer (18). 

Grimm et al. found that the serum level of vitamin D in patients with oral cancer was low; however, VDR was significantly more in premalignant lesions and oral SCC. They concluded that due to the increase in VDR in the patients, natural or therapeutic synthetic vitamin D can be used to induce an apoptotic effect in cancerous cells (27). Some studies stated that the metabolite concentration in the cells is responsible for anti-cancerous effects of vitamin rather than vitamin D serum level (22). However, the high prevalence rate of vitamin D deficiency in our control group and the high prevalence of cancer in Iranians should also be considered in interpreting the results.

In addition to the limitations mentioned earlier, one of the unexpected limitation in this study was Covid 19 pandemy and a significant increase in vitamin D intake and life style changes among all people,which made further sampling impossible. 

After this period, vitamin D levels cannot be compared with the previous data. In conclusion, the results of this preliminary study showed a high prevalence rate of vitamin D deficiency/insufficiency in patients with malignant SGTs and normal subjects. The serum level of vitamin D might not play a significant role in the pathogenesis of these tumors, similar to many human cancers. 

However, further prospective studies are recommended focusing on specific tumors and considering other factors, such as BMI, past long-term exposure, and long-term history of supplementary intake, exercise, skin complexion, sampling season, smoking habits, and parathyroid hormone levels.
